# Early literacy experiences constrain L1 and L2 reading procedures

**DOI:** 10.3389/fpsyg.2015.01446

**Published:** 2015-10-02

**Authors:** Adeetee Bhide

**Affiliations:** Department of Psychology, Learning Research and Development Center, University of PittsburghPittsburgh, PA USA

**Keywords:** orthographic depth hypothesis, whole word, phonics, ESL, word recognition

## Abstract

Computational models of reading posit that there are two pathways to word recognition, using sublexical phonology or morphological/orthographic information. They further theorize that everyone uses both pathways to some extent, but the division of labor between the pathways can vary. This review argues that the first language one was taught to read, and the instructional method by which one was taught, can have profound and long-lasting effects on how one reads, not only in one’s first language, but also in one’s second language. Readers who first learn a transparent orthography rely more heavily on the sublexical phonology pathway, and this seems relatively impervious to instruction. Readers who first learn a more opaque orthography rely more on morphological/orthographic information, but the degree to which they do so can be modulated by instructional method. Finally, readers who first learned to read a highly opaque morphosyllabic orthography use less sublexical phonology while reading in their second language than do other second language learners and this effect may be heightened if they were not also exposed to an orthography that codes for phonological units during early literacy acquisition. These effects of early literacy experiences on reading procedure are persistent despite increases in reading ability.

## Introduction

Models of word reading have broadly identified two pathways to word recognition: first accessing pronunciation or first accessing meaning. Everyone uses both pathways to some extent while reading, but the division of labor between them (i.e., *reading procedure*) can vary depending on the word type, context (Besner and Smith, 1992), the early literacy experiences of the individual, etc. In this review, I demonstrate that early literacy experiences have a profound effect on reading procedure. I begin by briefly reviewing differences between orthographies and models of word recognition. I then demonstrate the first language (L1) persistency effect, that effects of L1 orthographic transparency and instructional method are measureable, not only in beginning readers, but also in highly skilled adult readers. Finally, I demonstrate the L2 persistency effect, that that early literacy experiences are able to exert an effect even while one is learning to read a second language, and that these effects remain with increasing L2 proficiency. Although much work has been done on the effect of instructional method on reading procedure (Connelly et al., 2009) and on the effect of L1 transparency on both L1 (Katz and Frost, 1992) and L2 reading procedure, this review is unique in that it brings together these three lines of research into one integrated framework.

### Transparency

Writing systems are defined by the phonological grain size that each graph represents (see **Table 1**). Alphabetic orthographies, such as English, Serbo-Croatian, Korean^1^, Russian, German, and French, have graphs that code for phonemes. Alphasyllabic orthographies, such as Hindi, Marathi, and Thai, have graphs that code for syllables but subcomponents of the graphs code for phonemes. Abjads (e.g., Hebrew, Persian, and Arabic) are similar to alphasyllabaries but some^2^ vowel subcomponents are typically excluded in text. Syllabic orthographies, such as Japanese hiragana and katakana (collectively called kana), have graphs that code for syllables. Finally, morphosyllabic orthographies, such as Chinese and Japanese kanji^3^, have graphs that code for morphemes.

**Table 1:** The trade-off between phonology and morphology for the word “money” in various orthographies.

Orthography *Writing system* “money”	Graph-phonological unit consistency	Phonological unit-graph consistency	Trade-off between phonology and morphology (see Perfetti and Harris, 2013)
Japanese hiragana *Syllabary* 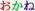 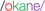	Most kana graphs have unique pronunciations, but there are exceptions (see Akita and Hatano, 1999)	Highly consistent	Precedence is given to preserving phonology
Spanish *Alphabet* 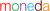 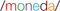	Fairly consistent but there are exceptions (see Defior and Serrano, in press)	Fairly consistent but there are exceptions (see Defior and Serrano, in press)	Precedence is given to preserving phonology; e.g., the “d” changes to a “t” in moneda → monetario (monetary)
Korean *Alphabet* 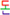 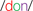	Highly consistent	Highly consistent	Phonological transparency has been sacrificed in a few words to make morphological relationships more apparent (see Perfetti and Harris, 2013)
Marathi *Alphasyllabary* 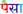 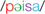	Highly consistent	Fairly consistent, but the visual forms of graphs change depending on orthographic context. The /ə/ is not always orthographically represented.	Precedence is given to preserving phonology; e.g., the 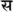 /s/ changes to a 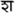 /ʃ/ in पैसा → 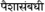 (monetary)
English *Alphabet* 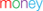 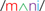	Inconsistent, especially for vowels	Inconsistent	Phonology is sometimes sacrificed for morphology; e.g., money and monetary both use the letter “o,” although its pronunciation changes
Hebrew *Abjad* 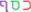 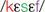	Highly consistent	Vowels are not orthographically represented. The visual forms of graphs can change based on word position.	Exclusion of vowels makes morphological relationships more apparent; e.g., money (which also means silver) shares a consonantal root structure with gray-haired (**כסוף שער**/kisuf se’ɒr/) and silver-plated (**הכספה**/hɒk^h^sɒfɒ/)
Chinese *Morphosyllabary* 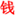 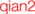	Typically consistent at multi-character level; Inconsistent at the character level; e.g., the phonetic radical in “money” can be found in characters that have various pronunciations such as jian1, jian4, pan4, can2, and zhan4	Extremely inconsistent: many characters represent the same syllable; e.g., 前,钳, and 乾 are all pronounced qian2	Phonological transparency is sacrificed for semantic information; e.g., the character for “money” has the semantic radical “gold.” Inclusion of semantic information distinguishes between homophones.


Phonological transparency refers to how systematically a given graph maps onto a given phonological unit and vice versa (see **Table 1**). Although languages with the same writing system can vary in terms of transparency, transparency is not independent of writing system. Alphabets range from highly transparent (e.g., Serbo-Croatian) to moderately opaque (e.g., English). Note that grapheme-phoneme and phoneme-grapheme consistencies are not necessarily equivalent; for example, French and German have higher grapheme-phoneme consistency than phoneme-grapheme consistency (Deacon et al., in press; Landerl, in press).

Alphasyllabaries have high graphemic subcomponent-phoneme consistency; graphemic subcomponents typically map onto only one phoneme. There is less phoneme-graphemic subcomponent consistency because the schwa vowel is inconsistently represented (Bhide et al., 2013). Furthermore, the visual form of a graphemic subcomponent may change depending on other subcomponents in the graph. Syllabaries tend to be highly transparent. The vowelized versions of abjads are highly transparent. In the unvowelized form, the grapheme-phoneme correspondences are highly transparent^4^. However, some vowel phonemes are not orthographically represented, so the phoneme-grapheme correspondences are highly opaque. Furthermore, the visual forms of graphs can change based on word position (Saiegh-haddad, in press).

**FIGURE 1:**
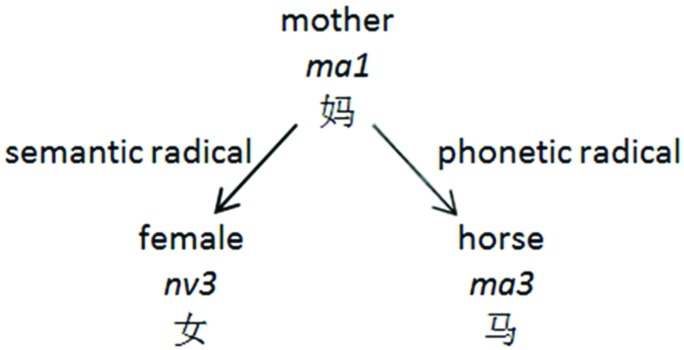
**An example of semantic and phonetic radicals within one character**.

In Chinese, a morphosyllabary, 80–90% of characters are made up of two components, a phonetic and semantic radical, which provide a cue to the character’s pronunciation and meaning, respectively (Kang, 1993 as cited in Li and McBride-Chang, 2014) (see **Figure 1**). However, a given phonetic radical can cue for multiple pronunciations and the same syllable can be cued by multiple phonetic radicals. Therefore, at the character level, morphosyllabaries are so highly opaque that they are considered an outlier orthography. However, multi-character, multi-syllabic words are highly transparent; each character typically has one pronunciation and represents one syllable in the word.

Opacity can stem from multiple sources: some phonemes may not be represented in the text (in the case of Hebrew) or multiple graphemes may correspond to the same syllable (in the case of Chinese). Readers of Hebrew must rely heavily on context to disambiguate the many homographs whereas readers of Chinese must have highly specified orthographic knowledge to distinguish between homophonic characters.

Opacity does not necessarily make an orthography more difficult to read. There is a trade-off between accurately representing phonological and morphological information and opacity often results from the inclusion of more morphological information at the expense of phonology [Perfetti and Harris (2013); see **Table 1**]. For example, the vowel digraph “ea” is pronounced differently in the words “heal” and “health,” making the words opaque. However, this phonological ambiguity makes the semantic similarity more apparent. The opacity also allows English to distinguish between homophones such as “heal” and “heel”. In abjads, such as Arabic, sequences of three consonants are used to form roots, which can be combined with a variety of vowels to form families of semantically related words. For example, the root k-t-b is found in the words ketaab (book), kataba (he wrote), and maktaba (library).

These 3-letter roots are more apparent in the unvowelized versions (Ryan and Meara, 1991), another example of how phonological opacity allows for more morphological clarity. In morphosyllabaries, the characters are highly opaque, but provide a great deal of semantic information via the semantic radical. The character system also allows for the disambiguation of the many homophones in Chinese and Japanese.

### Models of Word Reading

This basic division of labor can be simulated by computational models of word reading. The DRC model (Coltheart et al., 2001) models word naming and posits that there are two possible pathways: (1) the sublexical phonological route that uses letter-sound correspondence rules to sound out words and (2) a lexical route that maps the orthographic form onto a stored whole-word phonological representation. The pathways share a common speech output (phoneme) system called the response buffer^5^. If both pathways activate the correct phonemic sequence, the response is faster than if the pathways are in competition.

A connectionist alternative to the dual-route model is the triangle model (Seidenberg and McClelland, 1989). The triangle model was initially developed to model word naming, and also claimed that there are two pathways. The first pathway uses statistical regularities of sublexical constituents to activate phonological features, whereas the second maps the orthographic form onto its semantic features, and from there accesses the phonological features. Later, the triangle model was applied to examine how word meaning is computed (Harm and Seidenberg, 2004). Although there are differences between the models, both models posit that words can be read using either sublexical phonological information or morphological information derived from analyzing morphemes as orthographic wholes. This review examines the division of labor between the two pathways.

#### Assessing the Division of Labor

Although many different experimental manipulations can be used to examine reading procedure, there are seven *signature manipulations* that have been used in many studies. These *signature manipulations*, as well as other experimental manipulations/paradigms, are used throughout the review to examine reading procedure.

If the pattern of results outlined below are found in studies, it would suggest that people are heavily relying on the sublexical phonological pathway:

(1) *Lexicality: if words and non-words are named with the same speed and accuracy.* Words are read faster than non-words because words benefit from the use of both routes. If words do not show an advantage over non-words, it suggests that the morphological pathway is being underutilized during word reading (Frost et al., 1987).
(2) If large effects of length are seen.
  Because the DRC model claims that grapheme-phoneme correspondences are computed in a left to right fashion^6^ (Coltheart et al., 2001), longer words should take more time to decode via the sublexical phonological pathway.
(3) *Regularity: if readers are very slow to name exception words or make many regularization errors.* According to the DRC model, if the sublexical phonological pathway is heavily weighted while reading exception words, it will slow down the lexical pathway or produce regularization errors (Coltheart et al., 2001). According to the triangle model, the sublexical phonological pathway is slower and less accurate at naming exception words^7^ (Seidenberg and McClelland, 1989).
(4) *If homophone/pseudohomophone effects are seen.* According to the triangle model, the phonological pathway cannot distinguish between homophones, pseudohomophones, and exemplars (Harm and Seidenberg, 2004). Note that studies employing homophones and pseudohomophones use various paradigms (e.g., lexical decision, backward masking, text reading, definition selection, naming, semantic judgment, spelling recognition, etc.), but share an underlying theoretical logic.

In contrast, if the pattern of results outlined below is found in studies, it would suggest that people are largely using morphological/orthographic information:

(1) *If large frequency effects are seen in lexical decisions or naming.* According to the DRC model, entries in the orthographic lexicon are frequency-sensitive (Coltheart et al., 2001). Simulations with the triangle model demonstrated that the semantic pathway is more sensitive to frequency than the phonological pathway is (Harm and Seidenberg, 2004).
(2) *If large effects of imageability or semantic priming are seen during naming tasks.* Imageability is a semantic variable and semantic priming preactivates relevant semantic features (Katz and Frost, 1992; Harm and Seidenberg, 2004).

It is important to note that reading procedure is different than reading ability. Different reading procedures entail differing emphases on the two reading pathways. In contrast, reading ability refers to overall differences in reading accuracy and/or speed. This review focuses on reading procedure and hence does not examine whether particular orthographies or teaching methods are associated with higher reading ability. Rather, it examines whether particular orthographies or teaching methods are associated with different reading procedures, and to get a more pure measure of reading procedure, it primarily focuses on studies in which the samples are matched for overall reading ability. For example, differences in non-word reading accuracy are only meaningful when the groups are matched for word reading accuracy. Other measures of reading procedure, such as error type analysis, can give us some information about reading procedure even when reading ability is not matched, but the results should always be interpreted with caution.

## Reading in the L1

### Transparency of the L1 Orthography

Previous studies suggest that people whose L1s have transparent orthographies use more sublexical phonology while reading than do people whose L1s have opaque orthographies, and that this is true for both beginning and skilled readers. The studies have compared English to more transparent orthographies (e.g., Serbo-Croatian, German, Albanian, Greek, Japanese hiragana, Welsh) or to more opaque orthographies (e.g., Hebrew, Chinese, Japanese kanji). They used signature manipulations, such as lexicality, homophonic/pseudohomophonic status, length, frequency, and semantic priming, to examine reading procedure.

#### Beginning Readers

A comparison between 7 and 9 years old children learning German and English, matched for word reading skill, found that English-speaking children struggled more with reading non-words aloud than did German-speaking children. Because the children were also matched for years of schooling, this introduced age as a possible confounding factor (Wimmer and Goswami, 1994). However, the results are robust as another study was able to find the same effect using a different paradigm and without the age confound; Goswami et al. (2001) found that German-speaking children showed more pseudohomophonic interference and a greater length effect on a lexical decision task than did English-speaking children (matched for reading and spelling).

Other studies using larger age ranges (5–15 years old) and more diverse language groups (Welsh, Albanian, Greek, Japanese, and English) have corroborated the general pattern of results described above. These studies did not match the participants as well as the Goswami studies did, making the results of each individual study less convincing. However, taken as a whole, the research does suggest an effect of orthographic transparency on reading procedure. The studies found a stronger relationship between word length and naming time in readers of transparent as compared to opaque orthographies (Ellis and Hooper, 2001; Ellis et al., 2004). Furthermore, error types vary by orthography. Children reading transparent orthographies were more likely to make mispronunciations that result in non-words (e.g., saying “polical” for “political”), which is consistent with a phonological assembly strategy. In contrast, children reading opaque orthographies were more likely to make whole word substitution errors (e.g., saying “computer” for “complete”), which is consistent with a reading strategy that attempts to map the whole visual form onto a lexical entry (Ellis and Hooper, 2001; Spencer and Hanley, 2003; Ellis et al., 2004). However, as mentioned above, there are some problems with matching participants. Primarily, the participants in these studies were not matched for reading ability; children reading transparent orthographies had higher word reading scores than those reading more opaque orthographies. Hence, it is possible that these differences in reading procedures would diminish when using ability-matched samples. Furthermore, although the participants in the Ellis and Hooper (2001) and Spencer and Hanley (2003) studies comparing English and Welsh speaking students were relatively well matched demographically^8^, the participants in the Ellis et al. (2004) study comparing English, Japanese, Greek, and Albanian speaking students were not well matched in terms of recruiting and testing procedures as well as cognitive abilities.

#### Skilled Readers

In addition to the effects of orthographic transparency on beginning readers, as detailed above, effects of orthographic transparency have also been found among skilled readers, providing evidence for the L1 persistency effect. Readers of more transparent orthographies tend to read non-words more accurately and are less affected by lexicality on their naming reaction times (RTs) than are readers of more opaque orthographies. Furthermore, semantic primes during naming tasks benefit readers of more opaque orthographies to a greater degree than readers of transparent orthographies (Katz and Feldman, 1983; Frost et al., 1987).

Another way of measuring reading procedure is by forcing strategy changes. Frost et al. (1987) had undergraduate students perform a naming task with priming. In Hebrew, naming was significantly slowed when the prime was a non-word. A smaller effect in the same direction was found for English, but no effect was found for Serbo-Croatian. In another experiment, participants were asked to name words and non-words as quickly as possible. The authors varied the proportion of non-words within the lists. Hebrew speakers were significantly less accurate when there was a high proportion of non-words in the list, a trend in the same direction was seen with English speakers, whereas no effect was seen for Serbo-Croatian speakers. Non-word primes and high proportions of non-words force people to use the sublexical phonological pathway. Hebrew readers are accustomed to using the morphological pathway, and the forced strategy change slows them down. In contrast, Serbo-Croatian readers typically use phonological information, so no strategy change is needed.

The Frost et al. (1987) study demonstrated that readers of shallow orthographies use phonology to identify a word, whereas readers of opaque orthographies access phonology once a word is identified. Studies testing English and Chinese participants using a backward masking paradigm came to the same conclusion (Perfetti and Bell, 1991; Perfetti and Zhang, 1991; Perfetti et al., 1992). In the paradigm, a word target is briefly shown, followed by a brief prime, and then a pattern mask. The English-speaking participants were more accurate at identifying the word target when the prime was a pseudohomophone of the word than an orthographic control (e.g., more accurate at identifying “rate” in the condition rate-RAIT-XXXX than in the condition rate-RALT-XXXX). In contrast, priming the target character with a homophonic character did not improve the accuracy of the Chinese participants. The interpretation of these findings is that, in English, the partial products of target identification include pre-lexical phonology, so phonological primes are able to reinstate the partial products. In Chinese, there is no pre-lexical activation of phonology, so there is no effect of the phonological prime.

One possible problem with comparing the aforementioned English and Chinese studies is that the English speaking participants were on average younger (all undergraduates) than the Chinese speaking participants (mostly graduate students). Furthermore, the English tasks used pseudohomophone primes whereas the Chinese tasks used homophone primes. Another English-Chinese comparison used tighter age controls (all undergraduates) and homophones for both languages and also found that English speakers use phonology during word identification whereas Chinese speakers access phonology after word identification. However, in contrast to the studies cited about, this study looked at participants reading texts, rather than individual words. Feng et al. (2001) measured eye movements while participants read texts in which some words were either retained or replaced either by their homophone or an orthographic control (e.g., “creek” was replaced either by “creak” or “creed”). For the English texts, distributional analyses revealed that, for first fixations less than 200 ms, homophones were indistinguishable from the targets, both of which differed significantly from the orthographic controls. This was especially true if the homophone and the original word had a high degree of orthographic overlap and if the target was highly predictable from context. In contrast, when reading Chinese texts, participants had longer first fixation durations for all orthographic mismatches. This difference suggests that, while reading English, participants use phonology, orthography, and context to identify words. In contrast, while reading Chinese, people mainly use orthography.

### Instructional Method

In addition to orthographic transparency, instructional method can also influence reading procedure. There are numerous instructional philosophies for teaching literacy. Broadly, the instructional methods cluster into two groups; phonology-based methods (such as phonics) and semantics-based methods (such as the whole word method). Phonology-based methods all focus on sounding out words, although the grain size of focus can vary (see Brown and Deavers, 1999; Ziegler and Goswami, 2006; Asfaha et al., 2009; Nag, 2011; Kyle et al., 2013). In contrast, semantics-based methods focus on recognizing whole words and deriving meaning from text. The goal of this section is to compare phonology and semantics based methods to determine if they are associated with different reading procedures. I chose not to compare phonological methods that focus on different grain sizes on the assumption that they all foster a reading procedure that is more dependent on the phonological pathway. However, there is some preliminary evidence from an artificial orthography study that phonological methods focusing on larger grain sizes may foster reading procedures that rely more heavily on the morphological pathway as compared to phonological methods focusing on smaller grain sizes (Hirshorn et al., 2015). There is not enough current research to examine the effect of teaching phonology at different grain sizes on reading procedure in the present review, but it is an interesting area of further inquiry.

Almost all of the studies focusing on reading procedure as an outcome have compared the phonics and whole word instructional methods, so I discuss those in detail. In the phonics method, letter-sound correspondences are introduced in a systematic manner. The whole word method (or book experience in New Zealand), emphasizes communication and comprehension. Words are often memorized from texts proposed by students and letter-sound correspondences are not taught systematically. The majority of studies has been done with English, so I focus on English in this section and then examine how generalizable the conclusions are to other languages further on.

Studies were included in this section only if they compared people who learned English via the phonics and whole word methods and used signature manipulations to examine reading procedure. The signature manipulations used include lexicality, regularity, length, frequency, pseudohomophonic/homophonic status, and imageability manipulations. Many of these studies (see Connelly et al., 2009 for review) compared students from Scotland, whose curriculum stresses phonics instruction, and New Zealand, whose curriculum stresses book experience. Although these studies use a cross-national sample, Scotland and New Zealand have close cultural ties and their educational systems share a common history and educational culture. Therefore, differences found between the samples are likely due to instructional differences. In the systematic phonics approach in Scotland, individual letter-sound correspondences are explicitly taught and students are encouraged to use sequences of such correspondences to sound out unfamiliar words. In New Zealand, almost all of the literacy instruction centers on story texts. Teachers help students understand the meaning of the story, and teach them to recognize words using context cues, initial letters/letter clusters, and analogies to other words. However, they are never taught to sound out successive letters.

#### Beginning Readers

Studies with 6–8 years old children have shown that students taught with a phonics focus typically outperform students taught with a book experience focus on *non-word* reading tasks, even while controlling for word reading ability and various demographic factors such as age, years of schooling, socioeconomic status (SES), word recognition, aural vocabulary, spelling ability, and short term memory (Thompson and Johnston, 2000; Connelly et al., 2001; Thompson et al., 2008). In addition to finding differences in non-word reading accuracy, Connelly et al. (2001) also found differences for *word* reading accuracy. Specifically, children taught with a phonics focus named regular words more accurately, but children taught with a book experience focus named exception words more accurately. The authors also compared the children on their ability to name highly familiar words which they encounter daily (e.g., “the,” “he”). The children with phonics instruction read the words significantly slower than the children with the book experience instruction, suggesting that they were less successful at incorporating these words into their sight vocabularies. Connelly (unpublished thesis, as cited in Connelly et al., 2009) found a length effect in naming words with 5–6 years old children from Scotland but not from New Zealand (the children were matched for overall reading accuracy), again suggesting that children with explicit phonics instructions use more phonological recoding while reading.

Johnston and Thompson (1989) compared the performance of 7–8 years old children from Scotland and New Zealand (matched for age and reading ability, differences in vocabulary size were statistically controlled for) on a lexical decision task containing words, pseudohomophones, and non-words. The New Zealand children were equally accurate at rejecting the non-words and pseudohomophones, whereas the Scottish children were more accurate at rejecting the non-words than the pseudohomophones.

Finally, Connelly et al. (2001) found that children taught with a phonics focus are more likely to attempt to sound out unfamiliar words than are children taught with a book experience focus. The 6–7 years old children from Scotland were more likely to produce non-word errors or contextually appropriate errors that retained the pronunciation of at least two of the letters in the original word than were the children from New Zealand.

All of the English studies cited above used a cross-national sample to examine the effect of instructional method, so it is possible that there were some socio-cultural confounds. Two studies were able to minimize confounding factors by comparing instructional method within the same country. Foorman et al. (1991) compared first graders in the U. S. receiving either more or less letter-sound instruction in their school curriculums. The students were matched for reading ability, vocabulary, SES, and ethnic diversity. However, there were a couple of notable differences: the students receiving less letter-sound instruction were drawn from public schools and were on average 2 months older than the students receiving more letter-sound instruction, who were drawn from parochial schools. The authors found that the students receiving more letter-sound instruction showed larger regularity effects while reading aloud.

Whereas Foorman et al. (1991) compared students in the U. S., Landerl (2000) compared students living in England. The study was designed as a follow-up to the Wimmer and Goswami (1994) study cited previously. In the original study, children learning German were compared to children learning English to look for an effect of orthographic transparency. However, the children learning English were taught using a blend of the whole-word and phonics methods, whereas the children learning German were primarily taught using the phonics method. In the follow-up study, two groups of English-speaking children were used, one that received a mix of whole word and phonics instruction and one that received primarily phonics based instruction. They were compared to German-speaking children who received phonics based instruction. The three groups were equivalent in terms of their word reading. However, for non-word reading, the German-speaking children performed the best, followed by the English-speaking children receiving the phonics based instruction, whereas the English-speaking children receiving the mixed instruction performed the worst.

#### Skilled Readers

The studies cited above demonstrate that instructional method affects the reading procedure of children just beginning to read (5–8 years old), even while controlling for various confounding factors. However, the question that remains is, are these differences in reading procedure stable over time (i.e., can we demonstrate the L1 persistency effect)? It is possible that as reading skill increases, more words are added to sight vocabulary, reducing the need for sublexical phonology. Furthermore, as reading skill increases, all adults, despite how they were taught to read, may settle on the same “optimal” reading procedure. The research (reviewed below) suggests that this is not the case. The ability of specific tasks to detect differences in reading procedure fluctuates with age, but some effect of instructional method on reading procedure is measurable in both adolescence and adulthood.

Johnston et al. (1995) examined whether differences in reading procedure remain constant across adolescence. The participants included both 8 and 11 year-old children from Scotland and New Zealand who were matched on reading ability, vocabulary, chronological age, and ethnicity. They were tested on a lexical decision task that included words, pseudohomophones, and non-words. Unlike the 8 year-olds taught with the book experience method, the 8 year-old children taught with the phonics instruction were less accurate at correctly rejecting the pseudohomophones than the non-words. Both groups of 11-year olds were equally accurate at rejecting pseudohomophones and non-words. Therefore, for this task, the effect of instructional method diminishes with reading experience. The participants also completed a pseudohomophone sentence evaluation task, where they have to judge whether or not a sentence is orthographically correct. The incorrect sentences had one word replaced by either a pseudohomophone (e.g., Can you poast this letter?) or by a control non-word (e.g., She has loast her bag.). Both groups of 8 year-olds were less accurate at evaluating the sentences with a pseudohomophone than the sentences with a control non-word. The 11 year-olds taught with the phonics method (but not the 11 year-olds taught with the book experience method) were also less accurate at evaluating the sentences with a pseudohomophone. Therefore, for this task, the effect of instructional method becomes more apparent with age. Reading instruction also had an effect on the participants’ accuracy in a word-meaning task, where participants had to choose the correct definition for presented words. Some of the stimuli were homophonic (e.g., son) and their definition (e.g., child) and the definition of their homophone (e.g., light) were among the choices. Children taught with the phonics method made more errors when choosing the correct definition for the homophonic stimuli than did the children taught using the book experience method. Therefore, on this task, the effects of teaching methodology seem relatively stable during early adolescence.

Thompson et al. (2009) were able to find effects of instructional method even among skilled adults. They compared university students (matched for age and vocabulary) who had learned to read in Scotland and New Zealand on non-word reading. The non-words included irregular, body-consistent stimuli (e.g., thild) where two responses are legitimate, the regular response that is inconsistent with the bodies of all real words and the pronunciation that is consistent with the bodies of words such as “mild”. Although the two groups were equally accurate, the Scottish adults were more likely to give regular responses and less likely to give irregular responses than were the New Zealand adults. These results are easiest to explain using the DRC model. The sublexical route uses grapheme-phoneme correspondence rules to sound out a word, without taking into account the greater orthographic context. So, for “thild,” the sublexical route would read it as /𝜃cccld/. In contrast, the lexical route would activate orthographically similar words, such as “child,” “mild,” “thick,” and “third”. These lexical representations would activate their phonological representations which would then activate the phonemes within them. Therefore, /acccld/ and /𝜃/ would be highly active, producing /𝜃acccld/. Therefore, greater dependence on the sublexical phonological route produces the regular response, whereas greater dependence on the lexical route produces the irregular response. These data are harder to explain with the triangle model because the sublexical route uses statistical regularities that are sensitive to orthographic context. Therefore, the triangle model is more likely to produce irregular responses.

Another result from the same study allows us to more easily interpret reading procedure using both computational models. The participants were asked to name words that varied in terms of both frequency and imageability. The Scottish adults were more likely to make regularization errors while reading low frequency, low imageability words than were the New Zealand adults. These results suggest that, for Scottish adults, the word types that engage the lexical/semantic pathways to the smallest degree were unable to elicit enough support from the lexical/semantic pathways to avoid regularization errors.

### Summary and Extension to Other Languages

Overall, the research suggests that readers of transparent orthographies (at all levels of reading skill) heavily weight the sublexical pathway while reading. In contrast, readers of more opaque orthographies rely more on morphological/orthographic information. The research on this topic has compared English to more transparent alphabets and syllabaries (German, Serbo-Croatian, Welsh, Albanian, Greek, and Japanese hiragana) and to more opaque abjads and morphosyllabaries (Hebrew, Chinese, and Japanese kanji), and found that the general conclusion held for all of those orthographies. There appear to be no studies using alphasyllabaries, but we can predict that readers of alphasyllabaries heavily weight the sublexical phonology pathway because they are transparent.

Studies of English have suggested that, in addition to transparency, instructional method can also influence reading procedure. Students taught with a phonics-based focus more heavily weight the sublexical phonology pathway, whereas students taught with a book-experience or whole-word focus weight the morphological pathway more heavily. The question that remains is, is this conclusion generalizable to other languages, or is it English specific? The evidence reviewed below suggests that the conclusion is applicable to other opaque orthographies such as Chinese, but not to more transparent orthographies such as French.

Leybaert and Content (1995) compared two French-speaking schools in Belgium (matched for SES) that used different teaching methods and examined which pathway (sublexical phonological or morphological) was more heavily weighted in their students. The authors compared age and ability-matched samples in separate analyses to control for reading experience and reading skill, respectively. Four different reading tests were administered in which different variables were manipulated to tease apart the weightings given to different pathways. The first test manipulated word regularity^9^, the second test manipulated both complexity of the grapho-phonological correspondences^10^ and lexicality, the third test varied the frequency of words and the length of words and pseudowords, and the final test contained words, and homophonic and non-homophonic pseudowords. For all the reading tests, participants had to read the items aloud as quickly as possible.

When comparing the reading ability-matched groups, the effect of teaching methodology was only visible on one of these reading tests; on the test that varied both grapho-phonological complexity and lexicality, there was an interaction between teaching method and lexicality in that the students receiving whole word instruction were slightly more accurate on the words and less accurate on the non-words than the students receiving phonics instruction. When comparing age-matched groups, the fourth and sixth graders receiving phonics instruction showed a larger regularity effect on both RT and accuracy than those receiving whole word instruction^11^. Overall, the Leybaert and Content (1995) study showed a minimal effect of teaching methodology in developing readers; out of four reading tests intended to measure the weightings given to the two pathways, only one showed a significant effect of teaching methodology using age-matched groups and another using ability-matched groups. And, on the test that found effects using ability-matched groups, only a methodology x lexicality interaction was found, not a methodology x lexicality × graphophonological complexity effect. A three-way interaction was predicted because graphophonological complexity affects decoding, so its effect should be larger for non-words than for words only if participants heavily use the morphological pathway when reading words.

Instruction had relatively little influence in French, which has a transparent alphabet, but it had a significant effect for English, which has a more opaque alphabet. The results for Chinese, a highly opaque morphosyllabary, echo those of English: instruction is able to exert an effect. As stated previously, most Chinese characters contain two components, a phonetic and semantic radical (see **Figure 1**). Unlike the DRC model, which cannot handle Chinese due to its lack of grapheme-phoneme correspondence rules, the triangle model can be trained such that the sublexical pathway can learn the statistical regularities among the phonetic radicals, whereas the semantic pathway can learn the meaning of the whole character, with the help of the semantic radicals (Yang et al., 2006, 2008). Different instructional methods have been shown to favor one reading method over another.

In Hong Kong, Chinese is taught using the “whole word method.” Characters are taught through rote copying and in the context of texts. Children are encouraged to rapidly identify whole characters. In contrast, in Taiwan, teachers are more likely to draw attention to the phonetic radicals and to teach characters in phonologically related sets. Furthermore, an alphabetic system called Zhu-Yin-Fu-Hao is used to phonologically transcribe characters (**Table 2**) (Scholfield and Chwo, 2005).

**Table 2:** Several words written in traditional Chinese characters, as well as the alphabetic orthographies pinyin (used in Mainland China) and Zhu-Yin-Fu-Hao (used in Taiwan).

Character	Meaning	Pinyin	Zhu-Yin-Fu-Hao
媽	Mother	mā	ㄇㄚ
嗎	Dust (v)	mā	ㄇㄚ
膜	Film (thin covering)	mó	ㄇㄛˊ
辣	Spicy	là	ㄌㄚˋ


*Characters are able to distinguish between the homophones “mother” and “dust,” whereas the alphabetic orthographies are not.*

Scholfield and Chwo (2005) studied sixth grade students from Taiwan and Hong Kong whose schools were of similar sizes and served socioeconomically comparable populations. They presented two characters to the students and asked them to make a meaning similarity judgment. Some of the foils were phonologically similar, whereas others were graphically similar. The students from Taiwan were slowed to a greater extent by the phonologically similar foils, whereas the students from Hong Kong were slowed to a greater extent by the graphically similar foils. This finding suggests that the instructional method in Taiwan leads to a greater dependence on phonological recoding during character recognition.

The research so far suggests that instructional method has a greater effect on languages with opaque orthographies, such as Chinese and English, than on languages with more transparent orthographies, such as French. More studies are needed to confirm this general conclusion because, to the best of my knowledge, only one study has examined the effect of instruction on a transparent orthography. It is also possible that instruction may influence reading procedure when beginning to read a transparent orthography, but not after a critical level of fluency has been reached; some preliminary data with learning to read a transparent artificial orthography show an effect of instruction on reading procedure (Taylor et al., 2015). A review of the literature did not reveal any studies that have looked at the effect of instruction on learning to read an alphasyllabary, syllabary, or abjad. We can predict that, for transparent alphasyllabaries and syllabaries, teaching method should have little effect on the weightings given to the two pathways. In fact, because syllables are more salient than phonemes in spoken language (Ziegler and Goswami, 2005), instructional method may have less of an effect in transparent syllabaries than in transparent alphabets. Because abjads are opaque, I predict that teaching methodology should be able to exert an effect on reading procedure, but more research is needed to confirm this hypothesis. It is possible that other factors besides transparency [such as the size of the graphemic set, see Nag (2011)] could moderate the influence that instructional method has on reading procedure.

## Reading in the L2

The language one was first taught to read, and how he/she was taught, can have powerful effects on reading procedure well into adulthood. Furthermore, early literacy experiences can even affect one’s approach to reading in a foreign language. L1–L2 transfer effects have been broadly studied in the literature and evidence of transfer has been found at all the levels of the language system (MacWhinney, 2001). Although these broad transfer effects are outside the scope of this paper, I demonstrate that the transparency of one’s L1 orthography can have effects even while reading in a second language and these effects remain even with increasing L2 proficiency. Furthermore, the instructional method of L1 literacy may also affect reading procedure. Similar to the L1 research, the L2 research has largely focused on learning English. Therefore, I begin with the effect of L1 orthographic transparency and instructional method on learning to read English and then examine how generalizable the conclusions are to other languages later on.

### Transparency of the L1 Orthography

The studies included in this section have all compared L1 readers of morphosyllabaries to L1 readers of more transparent orthographies or to native English speakers. They have found that when L1 readers of morphosyllabaries learn a more transparent orthography, they use less sublexical phonology as compared to L2 learners whose L1 orthography is more transparent. This seems to be true for both intermediate and advanced L2 speakers. Although it is difficult to directly compare proficiency levels across studies, it is possible to roughly classify participants into intermediate and high proficiency categories (**Table 3**).

**Table 3:** Criteria used to classify participants as intermediate (italicized) or high **(bolded)** proficiency.

Study	TOEFL score	Time living in English-speaking country	No. of years studying English	Self-rated English fluency	Other
*Wang and Koda, 2005*	∼550 (PBT, post-1995)	<1 year	∼7	∼2/4	Michigan score: ∼63
*Wang et al., 2003*	∼560 (PBT, post-1995)	<1 year	∼8	∼2/4	Michigan score: ∼64
*Koda, 1990*			>6		∼17/31 on a cloze test (Bachman, 1982)
*Brown and Haynes, 1985*					Enrolled in English L2 classes; presumably low proficiency
*Koda, 1988*			>6		∼17/31 on a cloze test; ∼27/35 on a reading comprehension test
*Wade-Woolley, 1999*	∼50% accuracy on the vocab/reading comp section of TOEFL (PBT, pre-1995)	Japanese participants: ∼3 weeks in Canada; Russian participants: 4 years in Israel	∼9		Japanese participants in low-intermediate ESL classes; Russian participants identified by the University as having poor English skills; ∼79/106 on the Woodcock Word Attack Test
*Akamatsu, 2005*	Average scaled score of 52.1 on the vocab/reading comp section of TOEFL (PBT, pre-1995)	Most likely never lived in an English-speaking country			Currently undergraduate students in the English department of a Japanese university
**Wang and Geva, 2003**					∼40th percentile on PPVT
**Akamatsu, 1999**	80–100% accuracy (average scaled score of 63.8) on the vocab/reading comp section of TOEFL, (PBT, pre-1995)				Currently graduate students at a Canadian university
**Akamatsu, 2003**	>85% accuracy on the vocab/reading comp section of TOEFL (PBT, pre-1995)				Passed reading speed criterion in the Gray Oral Reading Test; currently graduate students or recent PhD graduates at a Canadian university
**Perfetti et al., 2007**					Described as fluent by the authors
**Tan et al., 2003**	>570 (PBT, post-1995)	3–7 years	∼12	∼4/5	


*The most widely used measure of proficiency is the Test of English Language Proficiency (TOEFL). The TOEFL has gone through four major revisions in recent history: (1) before 1995, there was a paper-based test (PBT). It had three sections (vocabulary/reading comprehension, listening comprehension, structure/written expression) (ETS, 1994; Encomium Publications, 2000). The vocabulary/reading comprehension section score could either be expressed as % accuracy or as a scaled score that ranged from 22–67 (ETS, 1994). (2) After 1995, a new PBT was introduced that had three sections (reading comprehension, listening comprehension, structure/written expression). The total scaled score ranged from 310–677 (Encomium Publications, 2000; ETS, 2014). (3) A computer based version was introduced in 1998 and (4) an internet-based version in 2005 (Wall and Horák, 2008).*

#### Intermediate Proficiency Readers

Studies of intermediate proficiency ESL readers (see **Table 3**) have focused on adult learners and used the signature manipulations of regularity, frequency, and homophonic/pseudohomophonic status to look at reading procedure. For example, Wang and Koda (2005) compared Chinese and Korean L1 participants on a naming task. Although the Chinese participants were older than the Korean participants, they were well matched in terms of English experience and proficiency. On the naming task, the Chinese L1 participants were less likely to regularize low frequency exception words than were Korean L1 participants. Furthermore, the Korean participants named non-words more accurately. Wang et al. (2003) studied a similar population, but on a different task. They presented participants with a semantic judgment task with four types of foils: similarly spelled homophones, similarly spelled controls, less similarly spelled homophones, and less similarly spelled controls. For example, for the category “type of weather,” the category exemplar was “rain,” the similarly spelled homophone foil was “rein,” and the similarly spelled control was “ruin.” For the category “breakfast food,” the category exemplar was “cereal,” the less similarly spelled homophone foil was “serial,” and the less similarly spelled control was “several.” Korean participants were more likely to make false alarms to homophone foils whereas Chinese participants were more likely to make false alarms to similarly spelled foils, suggesting that the Korean participants relied more on phonological information whereas Chinese participants relied more on orthographic information during the task (Wang et al., 2003).

Koda (1988) compared native Spanish, Arabic, Japanese^12^, and English speakers on a spelling recognition task (participants see a word and its homophonic foil and are asked to choose the correctly spelled word, e.g., rain, rane) and pseudoword selection task (participants see two non-words and are asked to choose the one that sounds like a real word, e.g., rane, tane). Although all participants were slower on the pseudoword selection task than the spelling recognition task, this difference was most pronounced in the native English speakers and Japanese L1 speakers. The participants also read two passages; in one all of the words were spelled correctly and in the second many words were replaced by their heterographic homophones (e.g., Ted and Bill went hiking in the mountains last weak). The native English speakers and Japanese L1 speakers were slowed to a greater extent on the passage with the heterographic homophones. Furthermore, when the participants were asked to go back and find the homophones, the native English speakers found more than the Japanese L1 speakers, who found more than the Spanish and Arabic L1 speakers.

The other studies examining reading procedure in intermediate level ESL participants have used non-signature manipulations to look at reading procedure. For example, Brown and Haynes (1985) found that, unlike Spanish and Arabic participants, there was no correlation between listening and reading comprehension in Japanese participants. These results suggest that the Spanish and Arabic participants were sounding out the words, and then using their listening comprehension skills to understand the text.

Koda (1990) found that, in contrast to Japanese participants, Arabic and Spanish participants^13^ were slowed down when they could not engage in phonological recoding. She gave the participants two passages which described five novel items. In one passage, the names were non-sense pseudowords whereas in the other passage the names were Sanskrit characters that were equally unfamiliar to all participants. The Arabic and Spanish participants were slowed down while reading the passage with the Sanskrit characters, presumably because they were unpronounceable and hence the participants had to rely solely on orthographic information to remember them. In contrast, the Japanese participants read both passages at approximately the same speed. These results can be best explained using the triangle model; if the Japanese participants are accustomed to mainly relying on the orthography to semantics pathway, the Sanskrit characters do not force them to change strategy. In contrast, if the Spanish and Arabic participants rely on phonology as well, the Sanskrit characters require a change in strategy.

Wade-Woolley (1999) found that Japanese adults outperform Russian adults on *confrontation spelling tasks*, where they have to decide which of the presented orthographic strings is the correct spelling for the auditorily presented word. This result suggests that the Japanese participants are more likely to read via stored holistic orthographic patterns. One problem with this study is that the participants were not well matched; the Russian participants had been living in Israel for an average of 3.9 years whereas the Japanese participants were in Canada for an average of 3 weeks. However, because the Russian participants had spent more time in an English-speaking country, one would expect them to outperform the Japanese participants. Because the opposite result was found, we can be fairly confident that differing levels of English experience were not confounding the results.

#### High Proficiency Readers

It is possible that as people gain proficiency in English, they adjust the weightings given to the two pathways and use a reading procedure more suitable for English’s level of transparency. However, studies using more skilled populations (see **Table 3**) have found no evidence of this and have instead supported the L2 persistency claim, that first language effects can be found even among advanced L2 speakers. Studies of more skilled ESL learners have used the signature manipulation of lexicality as well as unique behavioral paradigms and neuroimaging.

Wang and Geva (2003) compared second grade native Cantonese speakers who grew up in Canada (albeit lived in Cantonese-speaking communities) and began learning English in first grade (when they entered mainstream school) to their native English speaking peers. The groups were not well matched on cognitive measures; the native English speakers had higher vocabulary scores, but the native Cantonese speakers had higher non-verbal reasoning skills. Despite equivalent real word spelling skills, the native English speakers were better at spelling non-words. However, the native Cantonese speakers displayed superior orthographic skills. For example, the native Cantonese children outperformed their native English-speaking peers on a confrontation spelling task, even while controlling for non-verbal reasoning. They also showed higher performance in a task in which they have to reproduce briefly displayed pronounceable and non-pronounceable non-words from memory. Furthermore, the native Cantonese children were less affected by pronounceability, suggesting that they are less likely to use phonological recoding to help them remember the non-words^14^.

Akamatsu (1999, 2003) looked at the effect of visual distortion (cAse AlTeRnAtion) on word naming (1999) and passage reading (2003) among Persian, Chinese, and Japanese adults who were well matched in terms of English experience and proficiency. The Chinese and Japanese participants were slowed down to a greater degree by the case alternation than were the Persian participants. Interestingly, the effect of case alternation in the Akamatsu (1999) study was restricted to low frequency words. Because case alternation disrupts word shape cues, this finding suggests that L1 readers of morphosyllabaries strongly rely on word shape cues for all words, whereas L1 readers of alphabets mostly rely on word shape cues for high frequency words.

Up to this point, I have claimed that first language effects on second language reading are robust (i.e., they remain even with increasing second language proficiency) and used cross-study comparisons as evidence for this. However, this evidence is relatively weak, as different tasks were used in every study. One study was able to compare different proficiency levels on the same task and found no effect of proficiency, providing stronger evidence the L2 persistency claim. Akamatsu (2005) ran low proficiency Japanese–English bilinguals on the same task that he used in his 1999 study (which used high proficiency Japanese–English bilinguals) and compared the effect of case alternation on naming in both groups. There was no proficiency by case interaction for either RT or accuracy, suggesting that the effects of case alteration remain constant with increasing L2 proficiency.

Neuroimaging studies have confirmed that Chinese L1 participants tend to read in a “whole word” style, by demonstrating that, even while reading more transparent orthographies, Chinese participants use orthographic brain regions associated with reading morphosyllabaries rather than phonological brain regions associated with reading phonographic systems. For example, Perfetti et al. (2007) found that fluent Chinese–English bilinguals show bilateral activation in posterior visual areas when passively viewing words in both languages, whereas native English speakers show a left-dominant pattern. Tan et al. (2003) found that Chinese–English bilinguals strongly activate the middle frontal cortex when making rhyme judgments for both English and Chinese words. In contrast, the native English speakers used the inferior and frontal superior cortices to a greater degree when making rhyme judgments in English. Together, the results from these studies demonstrate that even when native Chinese speakers who are highly proficient in English are reading in English, they neurally process the visual input in a manner that is more similar to how they process Chinese, rather than how a native English speaker would process the same input.

### Instructional Method of L1 Literacy

Not only is the transparency of the L1 orthography important, but so is the instructional method of L1 literacy. In China and Taiwan, children are taught to read using alphabets known as pinyin and Zhu-Yin-Fu-Hao, respectively (**Table 2**), before being introduced to characters. In contrast, in Hong Kong, children are introduced to characters immediately (Wang and Geva, 2003). Characters are taught using a “look-and-say” method, where children are asked to memorize the meaning and pronunciations of characters, without the mediation of an alphabetic system (Holm and Dodd, 1996).

**FIGURE 2:**
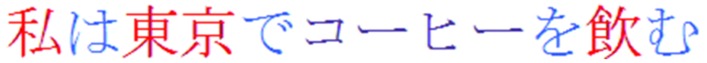
**This Japanese sentence means “I drink coffee in Tokyo” and is pronounced “Watashi wa Tokyo de koohii o nomu.” The red graphs are written in kanji, the blue graphs are written in hiragana, and the purple graphs are written in katakana.** The first kanji graph (red) means “I,” the second and third graphs mean “Tokyo,” and the fourth means “drink.” Note that the kanji characters are used for the content words. The first hiragana graph (blue) is a subject marker, the second is a location marker, the third is an object marker, and the fourth serves to conjugate the verb. The purple graphs mean coffee, pronounced “koohii,” a loan word from English.

Although alphabetic orthographies are sometimes used to teach Chinese, skilled adults only read characters. In contrast, skilled readers of Japanese must switch between three different orthographies (kanji, hiragana, and katakana) within the same text (**Figure 2**). Kanji is morphosyllabic and is typically used for content words. Hiragana and katakana (collectively called kana) are both syllabic. Hiragana is used to represent native Japanese words (such as participles and verb endings) and katakana is used for loan words. When learning Japanese, children learn to read all words (even content words that are typically written in kanji) using kana. Later, kanji characters are slowly introduced.

It is important to note that “differences in instructional method” has a much different meaning for alphabets than for morphosyllabaries. For languages with alphabetic orthographies (French, English), children are only learning one orthography, but the method by which they are taught to read that orthography differs in terms of how much phonics is included. In contrast, for languages with morphosyllabic orthographies (Chinese and Japanese), instructional method primarily refers not to how children are taught to read a given orthography, but to how many orthographies they are taught (although there may also be differences in terms of how much attention is drawn to phonetic radicals). Whether or not children are exposed to an orthography that codes for phonological units early in literacy acquisition may affect their reading procedure when they begin to learn a second language.

Studies examining the effect of instruction on L2 reading procedure have mainly relied on lexicality manipulations, although one non-signature experimental paradigm was used as well. The results from pseudoword reading tasks reviewed below suggest that accuracy differences between L1 readers of morphosyllabaries and alphabets can only be found if the readers of morphosyllabaries had no experience with a phonologically based orthography early in their learning. This may be why conflicting results have been found in the literature. For example, two studies have found no differences between participants with morphosyllabic and alphabetic backgrounds in terms of pseudoword reading accuracy (Koda, 1999; Wade-Woolley, 1999), whereas one study has (Holm and Dodd, 1996). Koda (1999) compared Chinese and Korean participants who were matched in terms of their TOEFL scores and Wade-Woolley (1999) compared Russian and Japanese participants who were matched in terms of their TOEFL scores and word reading. Both studies found that the two studied groups were equally accurate at pseudoword reading. In contrast, when Holm and Dodd (1996) compared students from Vietnam, Mainland China, and Hong Kong who were matched in terms of their real word reading and spelling abilities, they found that students from Hong Kong had significantly lower non-word reading accuracy than the other groups. In this study, a difference was found between students from Hong Kong and those from Mainland China, even though they both read morphosyllabic orthographies, likely because students from Mainland China were taught the alphabetic system of pinyin before they began instruction in Chinese characters, unlike the students from Hong Kong. Perhaps Koda (1999) and Wade-Woolley (1999) were unable to find significant effects because their participants had learned pinyin and kana, respectively, both of which code for phonological units.

Although pseudoword reading accuracy effects have only been demonstrated using participants who have no experience with writing systems that code for phonological units, RT effects have been found using Japanese participants (who have experience with phonologically based kana), perhaps because RT measures are more sensitive than accuracy measures. For example, Brown and Haynes (1985) found that Japanese participants showed a greater lexicality effect on their RT during a word/non-word reading task than either Spanish or Arabic participants did.

In addition to instructional effects on pseudoword reading, instructional effects have also been demonstrated on word reading tasks. Scholfield and Chwo (2005) asked Chinese–English bilinguals to make meaning similarity judgments in both Chinese (reviewed previously) and in English. They were shown two words, and had to judge whether or not they were semantically related. Some of the word pairs were phonologically similar (e.g., “right,” “write”), whereas others were graphically similar (e.g., “mother,” “bother”). They found that Taiwanese participants were both faster and more accurate on the graphically similar word pairs as compared to the phonologically similar word pairs, whereas the reverse was true for the Hong Kong participants. Although this data is suggestive of a difference in reading procedure, it is important to note that the Hong Kong participants had more weekly English lessons than did the Taiwanese participants. Their greater English fluency was reflected by their faster overall RTs to the English stimuli.

### Extension to Other Languages

In addition to the numerous studies done with English L2 learners, two studies have also looked at Japanese L2 learners. They have used both behavioral and neurocognitive measures and found that the results were consistent with English L2 studies. Chikamatsu (1996) compared English and Chinese L1 participants who were living in the U. S. and were in the same Japanese as a foreign language class. That class was their first introduction to Japanese, so all of the participants had the same educational experience, both in terms of spoken and written Japanese. Because they were beginners, they had only learned kana and had not been introduced to kanji. Participants performed a lexical decision task with three types of stimuli: familiar words (e.g., native Japanese words written in hiragana), unfamiliar words (e.g., loan words written in hiragana), and non-words. The results were consistent with the EFL research; Chinese L1 participants relied more on orthographic information and less on phonological information than did alphabetic L1 participants. The English and Chinese participants were matched for overall RT. However, the Chinese participants were slowed to a greater degree when switching from the familiar to unfamiliar condition than were the English participants, suggesting that they were using a visual-based strategy. Furthermore, the English participants demonstrated a stronger relationship between word length and RT than did the Chinese participants, suggesting that they were relying more heavily on phonological decoding.

Yokoyama et al. (2013) studied Chinese and Korean L1 readers who had studied Japanese for an average of 2.5 years. They found that when the participants performed a lexical decision task in their L2 orthography, Japanese kana, the Chinese L1 participants activated the left middle frontal gyrus more than the Korean L1 participants did. The left middle frontal gyrus is believed to phonological processor for morphosyllabic graphs. These results nicely dovetail with the ESL research, that L1 readers of morphosyllabaries use the same neural mechanisms when reading both their first and second languages.

The L2 research has demonstrated that L1 readers of morphosyllabic orthographies (Chinese, Japanese) tend to use less sublexical phonology than do L1 readers of more transparent orthographies (Korean, Russian, Spanish, Arabic, Persian, Vietnamese) while reading in their second language, especially if they were not introduced to a phonologically based orthography such as pinyin or kana during literacy acquisition. These effects can be explained by the assimilation/accommodation hypothesis (Perfetti et al., 2007), which states that people only change their reading procedure if necessitated by the properties of the L2 orthography. L1 readers of morphosyllabaries are accustomed to heavily weighting the morphological pathway, and because it is possible to read more transparent orthographies using the same reading procedure (even if it is not optimal), they will not change their reading procedure.

These first language effects are persistent; they can be found in beginning, intermediate, and advanced second language learners. The second language orthographies that have been studied are English and Japanese kana. The two orthographies are quite different; English has a moderately opaque alphabet whereas Japanese kana is a transparent syllabary. Because similar effects were found for both orthographies, we can predict that similar effects would be found if more transparent alphabets (e.g., Serbo-Croatian, French) or alphasyllabaries (e.g., Hindi, Thai) were studied as the second language. However, it remains unclear what effects would be seen if an abjad was chosen as the L2 orthography. Note the interesting difference between L1 and L2 learners: although instructional method has little influence on reading procedure when learning a transparent L1 orthography, language background is able to exert an effect on reading procedure when learning a transparent L2 orthography.

All of the studies reviewed above have compared L1 readers of morphosyllabaries to L1 readers of more transparent orthographies. An interesting area of future research would be to expand this research to other L1 groups. For example, I predict that L1 readers of English would show less reliance on sublexical phonology than would L1 readers of Spanish, German, Portuguese, and French when reading in Japanese kana, Greek, or Russian as their L2 orthography. There is limited evidence to support this hypothesis; Koda (1988) found differences in reading procedure between native English speakers and Spanish–English bilinguals. Furthermore, I hypothesize that English speakers from New Zealand may use less sublexical phonology while learning a second language than English speakers from Scotland because of the instructional differences in those two countries.

Some research has been done comparing L1 abjad (mainly Arabic) to other L1 groups learning English. However, the studies have primarily focused on Arabic speakers’ relatively poor word recognition skills, while controlling for other language skills (see Ryan and Meara, 1991; Fender, 2003, 2008b). Although this research is very interesting, it does not answer the main question of this review, specifically what reading procedure people use while reading. Therefore, it would be interesting to expand the work with Arabic L1 readers to look at reading procedure.

### Alternative Views

Although there is significant evidence that L1 readers of morphosyllabaries rely more heavily on the morphological pathway than do L1 readers of other orthographies while reading in their L2, there are some findings that do not neatly fit into that theory. For example, Akamatsu (1999) found that Chinese and Japanese participants showed a greater regularity effect on their RTs and that Chinese participants showed a greater regularity effect on their accuracy than did the Persian participants—the opposite of the expected result. Similarly, Wang and Koda (2005) found that Chinese participants showed a greater regularity effect on their accuracy than did Korean participants. Wang and Koda (2005) were able to account for this unexpected result by doing an error analysis: Korean participants were more likely to regularize irregular words than were Chinese participants. Akamatsu (1999) did not report error types so it unclear whether the same pattern holds for his study. Therefore, although the research broadly supports a difference in L2 reading procedure based on L1 literacy experiences, there are some anomalous findings.

Yamada (2004) suggested that first language influences on reading procedure may be due, not to the transparency of the orthography, but to the phonological properties of the language itself. If the L2 has a more complex phonological system than the L1, L2 learners may find it difficult to use sublexical phonology and therefore rely on morphological information. English is more phonologically complex than Chinese, which may be why L1 Chinese speakers use morphological information while reading in English. However, this explanation seems unlikely because differences were found between participants from Hong Kong and Mainland China on L2 English tasks, even though they both spoke a phonologically simple language. Furthermore, Chinese participants differed from both English and Korean participants while learning Japanese, which is also a phonologically simple language. Therefore, the orthographic transparency explanation seems to best account for all of the data.

As reviewed above, Scholfield and Chwo (2005) found differences between Taiwanese and Hong Kong students on an English word decision task. The authors acknowledged that these differences could be due to how Chinese is taught in the two countries (using Zhu-Yin-Fu-Hao in Taiwan and the whole word method in Hong Kong, the hypothesis that was espoused in this review) or to how English is taught in the two countries. During English instruction, Taiwanese schools focus on phonics whereas Hong Kong schools use the whole word method. It is impossible to know whether the differences in reading procedure stem from the manner of L1 or L2 literacy instruction in this case, because the two factors are confounded. The other study which found an effect of L1 instructional method on L2 reading procedure (Holm and Dodd, 1996) did not report on the English instructional methodology in the populations studied, so we do not know whether it was a confounding factor.

Studies examining the effect of L1 literacy *instructional method* on L2 reading procedure have not closely controlled for L2 literacy instructional method, making it impossible to know with certainty whether or not L1 literacy instructional method is sufficient to exert an effect on L2 reading procedure. It is also possible that some of the findings of L1 orthographic *transparency* were also confounded by L2 instructional method. For example, Wade-Woolley (1999) pointed out that, in Japan, English instruction is often very similar to kanji instruction; whole words are presented for memorization. However, some studies have successfully demonstrated the effect of L1 orthographic transparency on L2 reading procedure while controlling for L2 literacy instructional method. For example, Wang and Geva (2003) were able to control for L2 literacy instruction (but not English language experience) by comparing Cantonese–English bilinguals to their native speaking peers. In contrast, Chikamatsu (1996) was able to control for all aspects of L2 experience by comparing students who were in the same introductory Japanese class. Because significant differences in L2 reading procedure were found in both of these studies, it is clear that differences in L1 orthographic transparency are sufficient to affect L2 reading procedure.

## Conclusion

The division of labor between the sublexical phonological and morphological pathways can vary depending on word type, context, and the early literacy experiences of an individual. This review focused on variations across individuals and demonstrated that early literacy experiences, both which language one first learned to read and how one was taught to read, can have profound and long-lasting impacts on reading procedure. People who learn a more transparent orthography use more sublexical phonology while reading, whereas people who learn a more opaque orthography rely more heavily on morphological/orthographic information. For readers of more opaque orthographies (e.g., English, Chinese), instructional method also impacts reading procedure. These effects are measureable in both beginning and advanced readers.

Not only do early literacy experiences affect how one reads in one’s first language, they also affect how one reads in a foreign language. L1 readers of morphosyllabic orthographies use less sublexical phonology than do L1 readers of more transparent orthographies and these effects are measureable in beginning, intermediate, and advanced L2 learners of English and Japanese kana, in children and adults, and in comparison to participants with various L1 backgrounds. However, they may be moderated by whether or not a reader was introduced to an orthography that codes for phonological units (e.g., pinyin, Zhu-Yin-Fu-Hao, kana) during early literacy acquisition.

Although not the focus of this review, it is interesting to consider the clinical implications of differential reading procedures. For example, brain damage can selectively impair either the phonological or semantic pathway. The same patterns of brain damage may differentially affect the severity of reading impairment depending on the dominant reading procedure prior to injury. For example, damage to the semantic pathway would be less damaging in a person who primarily depended on the phonological pathway than someone who primarily depended on the semantic pathway prior to injury (Plaut et al., 1996). When assessing the impact of selective brain damage, it may be important to consider the person’s first language as well as their educational experiences.

It is important to note that that the majority of the conclusions in this review was drawn based on English, which in many regards has an outlier orthography. Where possible, research on other languages was included and hypotheses were made as to how applicable the conclusions drawn from English are to other languages. There is currently much more work being done with other languages, so hopefully some of the hypotheses made in this review can be empirically tested in the near future.

## Conflict of Interest Statement

The authors declare that the research was conducted in the absence of any commercial or financial relationships that could be construed as a potential conflict of interest.

## Footnotes

1 Unlike other alphabets, Korean uses a non-linear graph arrangement. Also, note that although Korean Hangul is alphabetic, Korean occasionally uses some Chinese characters as well (Taylor and Taylor, 1995).

2 In unvowelized Hebrew, almost all vowels are orthographically unexpressed. Two graphs can, in certain contexts, express vowels, however, these graphs also express consonants (Frost, 2012). In unvowelized Arabic, long vowels are orthographically expressed but short vowels are not (Fender, 2008a).

3 Note that Japanese kanji is not, strictly speaking, morphosyllabic. Most kanji characters have two readings, on and kun. The on-reading is the Chinese pronunciation whereas the kun-reading is the Japanese pronunciation. Although the on-reading is monosyllabic, the kun-reading may be multisyllabic. Furthermore, because kanji graphs have multiple pronunciations, there is low character-phonological unit consistency (unlike Chinese).

4 In Hebrew, some diacritical marks are used to distinguish consonants. These diacritical marks are excluded in the unvowelized form, making the consonants slightly ambiguous (Frost and Bentin, 1992).

5 Note that there are other dual route models which posit that all stimuli are processed by either the lexical or sublexical pathway and the pathways do not interact (see Norris and Brown, 1985) but there is considerable evidence against those models (Coltheart et al., 2001).

6 In linear alphabets that are read from the left to the right.

7 Especially low frequency exception words.

8 Ellis and Hooper (2001) matching criteria: the students all lived in the Wrexham area of Wales, their schools were all in similar in terms of catchment area, classroom size and teaching method, the students were all in Year 2, the students were matched in terms of math ability. Spencer and Hanley (2003) matching criteria: the students were in local education authority schools in Denbighshire, North Wales, the students were all in Year 2, the schools used phonics to teach literacy.

9 Regular words conform in pronunciation to the most frequent correspondences between speech sounds and letters/groups of letters. Irregular words include spelling patterns that deviate from their most common pronunciation.

10 Items with low graphophonological complexity have letters than only map onto one phoneme, independent of orthographic context. Complex items contain phonemes than can be represented by more than one letter or letters that can represent more than one phoneme depending on orthographic context.

11 Although some regularity effects were also seen with the second graders, these are difficult to interpret due to their low accuracy.

12 The EFL participants were matched on English ability as measured by cloze and listening comprehension.

13 All three groups were matched on English ability, as measured by a cloze test.

14 The main effect and interaction held even while controlling for non-verbal reasoning.
